# Effectiveness of Standard Oral Care Plan During Hospital Stay in Individuals With Brain Injury

**DOI:** 10.3389/fneur.2021.714167

**Published:** 2021-12-15

**Authors:** Simple F. Kothari, Gustavo G. Nascimento, Mille B. Jakobsen, Jørgen F. Nielsen, Mohit Kothari

**Affiliations:** ^1^Department of Clinical Medicine, Hammel Neurorehabilitation Centre and University Research Clinic, Aarhus University, Hammel, Denmark; ^2^Section of Orofacial Pain and Jaw Function, Department of Dentistry and Oral Health, Aarhus University, Aarhus, Denmark; ^3^Section of Periodontology, Department of Dentistry and Oral Health, Aarhus University, Aarhus, Denmark; ^4^JSS Dental College and Hospital, JSS Academy of Higher Education and Research, Mysore, India

**Keywords:** hospitalization, neurorehabilitation, nursing, oral health, oral hygiene, periodontitis, stroke, traumatic brain injury

## Abstract

**Objective:** To investigate the effectiveness of an existing standard oral care program (SOCP) and factors associated with it during hospitalization in individuals with acquired brain injury (ABI).

**Material and Methods:** A total of 61 individuals underwent a SOCP for 4 weeks in a longitudinal observational study. Rapidly noticeable changes in oral health were evaluated by performing plaque, calculus, bleeding on probing (BOP) and bedside oral examination (BOE) at weeks 1 and 5. Individuals' brushing habits, eating difficulties, and the onset of pneumonia were retrieved from their medical records. Association between oral-health outcomes to systemic variables were investigated through multilevel regression models.

**Results:** Dental plaque (*P* = 0.01) and total BOE score (*P* < 0.05) decreased over time but not the proportion of dental calculus (*P* = 0.30), BOP (*P* = 0.06), and tooth brushing frequency (*P* = 0.06). Reduction in plaque and BOE over time were negatively associated with higher periodontitis scores at baseline (coef. −6.8; −1.0), respectively, which in turn were associated with an increased proportion of BOP (coef. ≈ 15.0). An increased proportion of calculus was associated with eating difficulties (coef. 2.3) and the onset of pneumonia (coef. 6.2).

**Conclusions:** Nursing care has been fundamental in improving oral health, especially reducing dental plaque and BOE scores. However, our findings indicate a need for improving the existing SOCP through academic-clinical partnerships.

**Clinical Relevance:** Early introduction of oral care program to brain-injured individuals is beneficial in reducing plaque accumulation and improving oral health.

## Introduction

Oral care is essential to maintain oral health and prevent complications such as periodontal diseases and tooth loss in patients with acquired brain injury (ABI) (1–5). Poor oral hygiene among dependent hospitalized patients could lead to severe complications such as poor nutritional intake, increased length of hospital stays, and pneumonia (5–7). Concerning oral health, stroke can cause hemiparesis and hemiplegia to the facial muscles and the muscles of the pharynx, tongue, palate, and mastication, resulting in impaired oral clearance (8, 9). Medications prescribed for patients after stroke may further impact oral health resulting in, for example, dry mouth, oral ulcers, and stomatitis (10). Acquired brain injury individuals with swallowing difficulties have compromised oral clearance that may lead to increased bacterial load (5). Swallowing impairment, along with poor oral health has a significant impact on an individual's nutritional intake (11), increasing the risk of aspirational pneumonia (6, 12), which in turn has a negative impact on rehabilitation and other functional outcomes (6, 13).

Evidence suggests that stroke survivors with an increased plaque and bacterial load experience a deterioration of the periodontal conditions (1, 14). Recently, a study showed that 40% of the ABI population had an abundant amount of dental plaque and increased bleeding on probing (BOP), a finding that may indicate an acute hospitalization effect (2). In addition, 74% of the ABI individuals also had severe periodontitis, a condition, supported by their poor sociobehavioral and medical history, representing a chronic stage of an oral health disease (2).

Post ABI, many patients are reliant on nursing staff to assist them with oral hygiene. Despite indications that healthcare staff is interested in improving this aspect of care, a recent survey conducted with >250 health professionals showed that oral care had not been their prime focus due to barriers such as lack of time due to prioritizing other emergency tasks and unfocused oral care policies, and absence of training and evidence-based continuing education (15).

In the light of the current evidence on the importance of oral health among individuals with ABI, oral care management through oral care providers could play an important role in this area (11). It is not clear whether the existing oral care provided by healthcare professionals has any effect on the oral health of hospitalized patients with ABI in a neurorehabilitation setting (2, 15, 16). New knowledge on the topic may provide an overview to promote and manage oral health in these individuals. Accordingly, this study aimed to investigate the effectiveness of the existing oral care program over time (5 weeks) and its associated factors during hospitalization in patients with ABI. We hypothesized that the current standard oral care provision requires further structural improvement and modifications.

## Methods

### Participants and Recruitment

**Figure 1:**
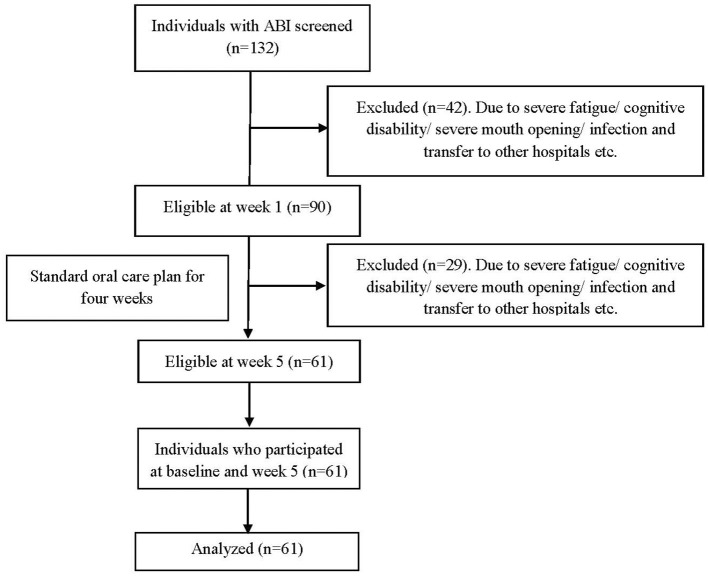
Flow chart.

All individuals with ABI admitted between February and June 2019 to the Hammel Neurorehabilitation and Research Centre (HNRC), Denmark, were recruited for this longitudinal observational study. Patients admitted reasons other than ABI were excluded, so were pregnant women. As some patients moved in and out from the HNRC due to medical emergencies or the need for other facilities unavailable at the center, they were included in the study if re-admitted within 5 days to the HNRC from their first day of admission. The readmission day was counted as their first day of admission. Individuals with ABI who prevented the examination for reasons such as fatigue/cognition/ limited mouth opening/stress/infection were rescheduled within 1 week and excluded from the study if it was not possible to re-examine within the week 1 window. In total, 132 individuals with ABI were screened and examined within the first week (baseline) from the admission day and later at week 5, re-examined to assess the acute changes in oral health (Figure 1). Out of 132 individuals, 90 were eligible for the week 1 assessment, and 61 individuals were eligible for the week 5 assessment after fulfilling all the above-mentioned eligibility criteria (Figure 1). The strengthening the reporting of observational studies in epidemiology guidelines were used to guide the reporting of the study.

### Procedures and Measures

#### Medical Records

Each individual's *main diagnosis, medical history, the onset of brain injury, and length of stay in acute care* were documented at week 1, and *onset of pneumonia during hospitalization*, clinically measured *body mass index*, eating difficulties, *dysphagia, and feeding status* were documented at weeks 1 and 5 from the e-journal of the patients (2, 3, 6).

#### Demographics and Sociobehavioral History

A structured questionnaire was used to elicit general and oral health-related social and behavioral history. The questionnaire elicited information on *age, sex, education level, profession, living status, smoking habits, and brushing and dental appointment frequencies*, which were recorded by a nurse (MBJ) at week 1 and in addition *brushing frequency during hospitalization* was again recorded at week 5 (2, 3, 6).

#### Motor and Cognitive Deficits Related to Orofacial Function Parameters

Motor and cognitive domains related to orofacial function were collected from the subset of the following brain injury scales at both baseline and at week 5: the early functional ability (EFA) scale (17), the functional independent measure (FIM) (18), the functional oral intake scales (FOIS) (19), and the Rancho Los Amigos scale (RLAS) (20).

##### Early Functional Ability Scale

The EFA scale (17) evaluates the early functional abilities in terms of basic everyday functions with increasing wakefulness and at the same time yet significant functional motor limitations. The scale comprises of 20 items, which are scored on a five-point Likert scale with 1 = “no function,” 2 = “severe disturbance,” 3 = “moderate disturbance,” 4 = “slight disturbance,” and 5 = “normal.” In this study, four items from EFA were taken as markers of orofacial dysfunction: (1) orofacial stimulation, (2) swallowing, (3) tongue movements/chewing, and (4) facial expressions/mimic.

##### Functional Independent Measure

Functional independent measure (18) assess ADL in patients with ABI 24, 25. Functional Independent Measure scale assesses motor and cognitive disability. The scale includes 18 items, of which 13 items are motor domains. Eating motor items were taken as markers of orofacial function whereas all five items of cognition, problem-solving, social interaction, comprehension, expression, and memory were included. Each item is scored from 1 to 7 based on level of independence, where 1 represents total dependence and 7 indicates complete independence.

##### Functional Oral Intake Scale

Functional Oral Intake scale (19) documents the functional level of oral intake of food and liquid in patients with stroke. This scale consists of seven items: tube dependent (levels 1–3): 1—no oral intake, 2—tube dependent with minimal/inconsistent oral intake, and 3—tube supplements with consistent oral intake 26; total oral intake (levels 4–7): 4—total oral intake of a single consistency, 5—total oral intake of multiple consistencies requiring special preparation, 6—total oral intake with no special preparation, but must avoid specific foods or liquid items, and 7—total oral intake with no restrictions.

##### Rancho Los Amigos Scale

Rancho Los Amigos scale (20) assesses the cognitive function in patients with post-coma. Patients are scored according to the levels: 1—response, 2—generalized response, 3—localized response, 4—confused, agitated response, 5—confused, inappropriate, non-agitated response, 6—confused, appropriate response, 7—automatic, appropriate response, and 8—purposeful, appropriate response.

In our previous study, by employing a factor analysis approach on the questionnaire data, we were able to identify two different factors, which were later dubbed as the “motor” domain based on their orofacial health and entire “cognitive” domain (6). Accordingly, a “motor” factor was defined based on the scores of the eating domain of the FIM questionnaire, the total score of the FOIS questionnaire, and the orofacial stimulation, and swallowing domains of the EFA questionnaire. The “cognitive” factor, comprised the total score of cognitive FIM which included, problem-solving, social interaction, comprehension, expression, and memory domain, and the total score of the RLAS questionnaire (6). As the tongue and mimic domains of the EFA questionnaire loaded in both the factors, they were not included in either of the factors (6).

#### Comprehensive Oral Health Assessment

The clinical oral examination was conducted by a trained dentist (SFK) during week 1 and week 5 of hospitalization. This examination consisted of (1) *BOP examination:* was performed at six sites of each tooth (starting from the distal to the mesial end of each tooth buccally and palatally/lingually, respectively) by tipping a University of North Carolina-15 periodontal probe (PCPUNC15, Hu-Freidy, Chicago, IL, USA) until light resistance offered by the gingival tissues could be perceived (21). Bleeding on probing was recorded as absent or present. (2) *Plaque, and calculus detection:* was performed at six sites of each tooth using the tip of the periodontal probe at the dentogingival junction as recommended by O'Leary et al. (22, 23). Each condition was recorded as a dichotomous variable, based on its presence or absence. (3) *Bedside Oral Examination (BOE):* After permission from the author, *BOE* parameters were included (24). It has eight categories and three numerical and descriptive ratings (1—normal; 2—moderate dysfunction; and 3—severe dysfunction). Total BOE scores ranged from 8 (excellent oral health) to 24 (poor oral health). Bedside oral examination scores ranging from 8 to 10 are considered as indicative of excellent oral health, from 11 to 14 as moderately impaired oral health, and from 15 to 24 as significantly impaired oral health (24).

In addition, at baseline, we also assessed probing depth, gingival margin level, and clinical attachment level (CAL) with the use of the aforementioned probe. The baseline periodontal data was also submitted to factor analysis, which allowed us to identify two periodontal phenotypes, dubbed as “moderate” (number of sites with CAL = 3 or 4 mm, Periodontal Pocket Depth (PPD) = 3 or 4 mm, and the number of sites with BOP) and “severe” (number of sites with CAL ≥ 5 mm, the number of sites with PPD ≥ 5 mm, and the number of sites with suppuration) periodontitis. Detailed information about the identification of the periodontal phenotypes using the baseline periodontal data can be found elsewhere (2, 4).

#### Standard Oral Care Program at HNRC

Based on the Danish national clinical guidelines for oral care, the healthcare professionals follow the standard oral care program (SOCP) in all the individuals admitted at HNRC (25). The SOCP was supplemented by additional oral care depending on the individual needs. See Table 1 for details.

**Table 1:** Standard oral care program at HNRC.

**Individuals at HNRC**	**Standard oral-care (recommended clinical guidelines)**	**Supplemental oral-care (case-dependent)**
All ABI individuals **(self-oral care)**	Instruction to brush twice a day, preferably after each meal. Free to have any toothbrush they bring from home (small head/big head/electric soft bristle toothbrush) with fluoride toothpaste (min. 1,450 ppm).	Chlorhexidine mouth wash (0.12%). Oral mucosal care. Dental floss once a day. Lip moisturizer for dry or cracked lips.
ABI individuals with eating or cognitive difficulties **(oral care by caregivers)**	Orofacial stimulation (face, lip, gum, and tongue) before every meal. Cleaning of mouth for food debris and secretions before and after each intake of food and drinks. Use of small head soft bristle toothbrush and fluoridated non-foaming toothpaste (min. 1,450 ppm). Tooth brushing in circulatory motion starting bucally, palatally, and then to occlusal table twice a day after each meal.	Chlorhexidine mouth wash (0.12%). Oral mucosal care. Lip moisturizer for dry or cracked lips.
Tracheotomized ABI individuals **(oral care by caregivers)**	Oral care in recline or side wise position. Orofacial stimulation (face, lip, gum, and tongue) 2–3 times/day. Cleaning of mouth for food debris and secretions 2–3 times/day. Individual who are unable to spit, the oral cavity is cleaned by mouthwash having amyloglucosidase and fluoride (Zendium) with the help of sponge or swabs.	Carbonated water for patients with dry mouth. Mouthwash containing both chlorhexidine (0.05%) and fluoride (0.05%) for bleeding gums. Lip moisturizer for dry or cracked lips.

### Data Analyses

Data on the proportion of plaque, calculus, BOP, BOE scores, and frequency of tooth brushing were submitted to descriptive analyses. In addition, paired analyses (*t*-test for normally distributed variables and Wilcoxon signed-rand test for non-normally distributed variables) were also conducted. Using multilevel mixed-effects regression models, we were able to investigate the association between changes in oral health outcomes (proportion of plaque, calculus, BOP, and BOE scores) with both time-varying, collected at both baseline and week 5, and non-varying (elicited at baseline only) variables (sociodemographic and behavioral factors, systemic diseases, and motor and cognitive deficits related to orofacial function parameters). Variable selection was performed using the “backward” stepwise procedure, in which all variables were entered in the model, and then subsequently removed. Only variables with a *P*-value <0.20 were maintained in the model and those with a *P*-value <0.05 were considered statistically significant. The data analysis was carried out using the software Stata 14.2 (StataCorp., College Station, TX, USA).

## Results

Of the 90 patients included at baseline, 61 provided data for the 5-week follow-up (Figure 1). The mean age was 55.1 years (±14.0), and 64% of patients were male. More information about the sociodemographic data of the participants can be found elsewhere (2). A *post-hoc* sample size power revealed that using the BOP and plaque data at baseline and week 5 and assuming a correlation between the paired estimates of 0.7, our sample reached a power of 81%.

Paired analysis revealed that the proportion of sites with visible dental plaque (*P* = 0.01) significantly decreased over time but BOP (*P* = 0.06), calculus (*P* = 0.30), and the frequency of tooth brushing (*P* = 0.06) did not achieve statistically significant changes after 5 weeks of hospitalization. In addition, the total BOE score (*P* < 0.001) significantly improved over time, and most of the BOE domains like, swallow, saliva, mucosa, teeth, and odor (*P* < 0.05) (Table 2).

**Table 2:** Changes in oral health parameters during hospitalization.

**Items**	**Baseline** **(Week 1)**	**Week 5/Discharge**	***P*-value**
***1. % Plaque^*b*^***	50.6 (27.1)	42.2 (30.4)	0.01
***2. % BOP** ^***b***^*	41.9 (42.7)	30.4 (37.3)	0.06
***3. % Calculus** ^***b***^*	5.5 (12.5)	4.7 (11.9)	0.29
***4. BOE** ^***a***^*			
a. Swallow	1 (1–2)	1 (1–1)	<0.01
b. Lips	1 (1–2)	1 (1–1)	0.10
c. Tongue	1 (1–2)	1 (1–2)	0.29
d. Saliva	1 (1–2)	1 (1–2)	0.03
e. Mucosa	1 (1–2)	1 (1–1)	<0.01
f. Gingiva	1 (1–2)	1 (1–1)	0.07
g. Teeth	1 (1–2)	1 (1–1)	<0.01
h. Odor	1 (1–2)	1 (1–1)	0.05
***Total***	11 (9–13)	9 (8–11)	<0.001
**5. Frequency of toothbrushing** ^a^	1.8 (0.6)	2.0 (0.5)	0.06

*BOE, bedside oral examination; BOP, bleeding on probing*.

a *Wilcoxon signed ranked test*.

b *Paired t-test*.

Mixed-effects regression models indicated that individuals with “moderate” periodontitis at baseline (coef. −6.8) and those hospitalized at the regional ward (coef. −15.6) had decreased proportion of sites with dental plaque. In addition, the number of extracted teeth (coef. −1.0), the proportion of calculus (coef. −0.5), and the time (coef. −8.8) were also associated with a reduction in the proportion of plaque (Table 3A).

**Table 3A:** Mixed effect regression model comparing plaque and systemic findings.

	**% Plaque**		
**Variables**	**Coefficient (β)^a^**	**95% CI**	***P*-values**
“Moderate” periodontitis at baseline	−6.8	−12.1; −1.5	0.012
Regional ward (Reference: high-specialized ward)	−15.6	−27.3; −3.9	0.009
# Extracted teeth at baseline	−1.0	−1.9; −0.1	0.05
% Calculus	−0.5	−0.9; 0.0	0.032
Time	−8.8	−15.4; −2.2	0.009

a *Adjusted for age, BMI, and 'severe' periodontitis at baseline and variables in the model*.

Patients with higher scores of both “moderate” (coef. 14.3) and “severe” (coef. 15.6) periodontitis at baseline had an increase in the proportion of sites with BOP over the study period (Table 3B), whereas those who improved their “cognitive” domain (coef. −6.6) had a decrease in the proportion of BOP. The number of extracted teeth at baseline (coef. 0.5) and increased proportion of plaque over the study period (coef. 0.4) were also associated with an increased proportion of BOP after the 5-week follow-up. As displayed in Table 3C, those who developed pneumonia during hospitalization (coef. 6.2) and those with eating difficulties over the study period (coef. 2.3) had an increase in the proportion of sites with dental calculus.

**Table 3B:** Mixed effect regression model comparing BOP with systemic findings.

	**% BOP**		
**Variables**	**Coefficient (β)^a^**	**95% CI**	***P*-values**
“Moderate” periodontitis at baseline	14.3	9.6; 19.0	<0.001
“Severe” periodontitis at baseline	15.6	11.6; 19.5	<0.001
# Extracted teeth at baseline	0.5	0.1; 1.1	0.045
“Cognitive” domain over the study period	−6.6	−11.6; −1.6	0.010
% Plaque	0.4	0.2; 0.7	0.001

a *Adjusted for #Decayed teeth, FIM scores and variables in the model*.

**Table 3C:** Mixed effect regression model comparing calculus with systemic findings.

	**% Calculus**		
**Variables**	**Coefficient (β)^a^**	**95% CI**	**P-value**
Onset of pneumonia during hospitalization	6.2	1.4; 9.9	0.009
Eating difficulty	2.3	0.6; 4.0	0.007

a *Adjusted for ward, age, 'Cognitive' domain over the study period and variables in the model*.

Finally, mixed-effects regression models indicated that the individuals with higher scores of “moderate” periodontitis at baseline (coef. −1.0), those hospitalized at the regional ward (coef. −1.7), and those who improved their “motor” skills during the study period (coef. −0.6) had a reduction in their total BOE score, whereas those with dysphagia at baseline (coef. 0.5) and the old individuals (coef. 0.04) had an increased total BOE score after 5 weeks (Table 3D).

**Table 3D:** Mixed effect regression model comparing BOE data with systemic findings.

	**BOE—total score**		
**Variables**	**Coefficient (β)^a^**	**95% CI**	***P*-value**
“Moderate” periodontitis at baseline	−1.0	−1.3; −0.5	<0.001
Regional ward (Reference: high-specialized ward)	−1.7	−2.8; −0.5	0.006
“Motor” domain over the study period	−0.6	−1.0; −0.1	0.010
Dysphagia at baseline	0.5	0.1;1.1	0.043
Age	0.04	0.0;0.1	0.016

a *Adjusted for variables in the model*.

## Discussion

The main finding of the study was that the oral health parameters such as visible plaque and BOE scores significantly improved during a 5-week stay at neurorehabilitation setting following the current SOCP. Although a reduction in the proportion of sites with BOP and frequency of tooth brushing over time (5-week stay) was observed, it did not reach statistical significance. These findings demonstrate that although there was an improvement in the oral health status in hospitalized individuals, it was not substantiated, indicating a need for further development in the oral care program.

A significant reduction in the amount of dental plaque was observed over time. Dental plaque is a biofilm that comprises a diverse community of microorganisms formed regularly on the tooth surface and can be disrupted with proper toothbrushing and interdental cleaning (5, 26, 27). Individuals with less severe ABI are usually admitted to the regional ward at HNRC instead of the highly-specialized ward due to their better motor and cognitive functions, which makes them more co-operative than severely affected individuals with ABI (28). It was also evident from the mixed regression analysis that individuals admitted to the “regional ward” showed a strong association in reducing plaque compared to individuals from the highly specialized ward (25). This finding indicates that the poorest oral health conditions and the least oral health improvements occurred in moderate and severe ABI cases, i.e., those requiring most caregivers' attention. Hence, it is of utmost importance to properly train these professionals to improve oral health and, consequently, the quality of life of individuals with ABI. Interestingly, the proportion of plaque was also reduced in individuals with “moderate” periodontitis and with an increased proportion of calculus and BOP, indicating that there are also other factors such as the host immune response (6), which were not taken into account in this study, that might have influenced BOP.

Bleeding on probing is a sign of inflammation that occurs as a response to plaque accumulation on the periodontal tissues (29). In general, good oral hygiene practices are sufficient to control and reduce gingival bleeding (24, 27), which was also shown in the current study with a strong association between plaque and BOP (Table 3C). However, despite the significant plaque reduction, the proportion of sites with BOP did not reduce in the same individuals over the study period, as it probably originated from deep pocket rather than from the gingival tissues. It is also important to discuss that the SD values of BOP were probably quite high as few individuals had a very low BOP, while some (especially those with periodontitis) accumulated most of the BOP burden. Such a finding suggests that factors other than plaque might play a role in the onset and progression of gingival inflammation. It has been shown that a more exacerbated and rapid immune response, acute hospitalization, and cognitive and systematic complications are linked to a higher neutrophilic activity, which mounts an immediate gingival inflammatory response when exposed to plaque (30–32). Interestingly, our findings demonstrated that BOP decreased over time in individuals who showed an improvement in their “cognitive” function, indicating a reduction in confusion and agitation leading to increased cooperation with oral care, which very well-correlates with previous research (6). It has been shown that BOP is closely associated with “severe” periodontitis, which in addition to an already existing cognitive impairment, may contribute to other chronic conditions that share a common biological background to ABI (6, 33). Assuming that such an exacerbated immune response is not restricted to the oral cavity, this may interfere with other inflammatory processes, especially in a hospital setting and in the presence of other comorbidities, explaining partially our findings (6). On the other hand, the proportion of sites with BOP increased over time among patients with both “moderate” and “severe” periodontitis, despite the increase in toothbrushing frequency over the same period. This finding indicates that the oral health status in these patients was poor and tooth brushing alone may not be enough to tackle periodontitis, which can only be treated using scaling and root planning performed by dental professionals. In addition, toothbrushing is unable to remove calculus, a factor that contributes to further plaque accumulation, inflammation of gingival tissues, and progression of periodontitis. Furthermore, brushing is also thought to be more optimal for cleaning facial surfaces of teeth compared to interproximal/interdental surfaces that present a higher risk of plaque accumulation and developing periodontal lesions (34). Thus, interdental cleaning aids such as dental floss, and interdental brushes may prove to help decrease BOP as interdental cleaning has shown to be associated with less plaque, calculus, and gingivitis (35). Nevertheless, despite the efforts made by nurses to maintain oral hygiene, there was still deterioration of the inflammatory periodontal condition (5). This suggests a need for the involvement of dental personnel in hospitals for providing adequate oral care to patients with ABI (2).

Calculus, defined as hard deposit around the gingiva as a result of long-term plaque accumulation, showed no significant improvement over time, indicating that the amount of calculus identified was already present when the individuals were hospitalized. It is important to highlight that calculus does not indicate disease, but it makes oral hygiene more difficult to maintain and works as a plaque-retaining factor (36). It is known that periodontal pockets can be the focus of infection and calculus removal can improve the clinical condition of patients and reduce the length of hospital stay (37). Even though the removal of calculus is not possible without professional dental assistance, it is possible to maintain proper oral hygiene by preventing calculus formation. Such a finding supports the idea that chronic oral changes require professional help from dental personnel and changes in sociobehavioral factors for the improvement of oral health (29).

Our findings also revealed that individuals with eating difficulty and those who developed pneumonia during hospitalization had an increase in the proportion of sites with dental calculus. One may speculate whether the combination of dental calculus and eating difficulties may influence the onset of pneumonia. A recent study on patients with ABI has shown a robust association between periodontitis and debilitating conditions like dysphagia, dependency on a feeding tube, which is a major concern, as they lead to pneumonia (6). Although our study does not allow us to disentangle the causal relationship between these conditions, our overall findings suggest the need for increased focus on oral care especially for ABI individuals with conditions like eating difficulties and severe cognitive disturbances.

Interestingly, BOE scores decreased in individuals with higher scores of “moderate” periodontitis. As discussed, “moderate” periodontitis originates essentially from neglected oral hygiene, so do most of the BOE domains (2, 6, 24, 38). Thus, the combined effect of plaque reduction and increased frequency of oral hygiene can explain this association. It should be noted that, although BOE is a simple and easy-to-use tool in hospital settings, especially in intensive care units, its usefulness is questioned in patients with ABI, and therefore, the BOE results may be carefully interpreted (2, 4). This is because the instrument seems not to reflect the real clinical conditions of patients with ABI, thus, affecting the treatment plan. Furthermore, it has been shown that “aging” patients have more compromised function than young individuals, making them more vulnerable to dysphagia and unable to perform and maintain good oral hygiene procedures (2, 6).

A recent survey conducted among 157 oral caregivers at HNRC showed that the majority of oral caregivers were aware of the existing “Danish National Clinical Guidelines for Oral Care” (25). However, a significant number of oral care providers did not follow the guidelines systematically, expressing it as ineffective, time-consuming, and difficult to follow (15). Professionals were aware that patients with eating difficulties have challenges and different requirements (15) and on top, cognitive, and motor deficits add an extra challenge to oral hygiene maintenance (6). In addition, there is always a professional dilemma to maintain oral hygiene standards whilst respecting the autonomy of patients once they refuse oral hygiene care, even if it is required. Therefore, all these factors should be considered while formulating and designing oral care training and guidelines to improve oral care in a neurorehabilitation setting.

### Methodological Considerations

The current study sample originates from a single hospital setting, and therefore, our findings may have limited external validity. However, it is worth mentioning that this hospital is a reference center for the treatment of patients with ABI and receives patients from most regions of Denmark. In addition, a limited sample size and 30% lost to follow-up might have reduced the analytical power, as can be noted by borderline *P*-values. However, as aforementioned, our sample reached a power of 80%, which can be considered an acceptable value for this study. Future studies with large samples originating from several centers are needed. Another limitation of the study was the short follow-up time, given the chronicity of the most common oral diseases, i.e., dental caries and periodontitis. However, treatment of these conditions demands the involvement of dental personnel with appropriate armamentarium, which was not within the scope of the study. As our purpose was to observe the effect of an existing oral care program during hospitalization on oral health, we decided to evaluate conditions such as the proportion of dental plaque and BOP, as those parameters can rapidly change. We also need to be aware that few patients were excluded due to extreme fatigue, agitation, motor-cognitive deficits, leaving us with no opportunity for clinical examination, which might be a bias in representing the entire oral health status. Finally, since different oral care measures were implemented depending on the patients' condition, the distinct levels of care might have impacted our results. However, our main goal was to evaluate whether the standard oral care plan delivered during hospitalization was effective to improve oral health rather than to evaluate the most effective plan. Hence, further studies are needed to elucidate this aspect.

## Conclusions

A significant reduction in dental plaque and total BOE score was observed over time. However, non-significant improvements in gingivitis, the proportion of calculus, and brushing frequency indicate the need to further develop oral care programs for individuals with ABI keeping motor-cognitive deficits and eating difficulties in consideration. This study also enforces the need for the involvement of dentists in educating and supervising non-dental professionals at an early stage to provide a better and integrated oral care program for ABI individuals in hospital settings.

## Data Availability Statement

The raw data supporting the conclusions of this article will be made available by the authors, without undue reservation.

## Author Contributions

JFN and MK: conceptualization and methodology. SFK, GGN, and MK: validation and writing—original draft preparation. GGN: formal analysis. MBJ and SFK: investigation and data curation. SFK, GGN, JFN, and MK: writing—review and editing. MK: resources, visualization, supervision, project administration, and funding acquisition. All authors have read and agreed to the published version of the manuscript.

## Funding

MK received grant from Health Research Fund of Central Denmark Region for the current study with grant number A1407. The authors' salary was supported by their respective institution.

## Conflict of Interest

The authors declare that the research was conducted in the absence of any commercial or financial relationships that could be construed as a potential conflict of interest.

## Publisher's Note

All claims expressed in this article are solely those of the authors and do not necessarily represent those of their affiliated organizations, or those of the publisher, the editors and the reviewers. Any product that may be evaluated in this article, or claim that may be made by its manufacturer, is not guaranteed or endorsed by the publisher.
